# Fish Consumption during Pregnancy, Mercury Transfer, and Birth Weight along the Madeira River Basin in Amazonia

**DOI:** 10.3390/ijerph10062150

**Published:** 2013-05-28

**Authors:** Rejane C. Marques, José V. E. Bernardi, José G. Dórea, Katiane G. Brandão, Lucélia Bueno, Renata S. Leão, Olaf Malm

**Affiliations:** 1 Federal University of Rio de Janeiro, Campus Macaé, CEP 27930-560, RJ, Brazil; E-Mail: rejanecmarques@globo.com; 2 University of Brasília, Brasília, CEP 0919-970, DF, Brazil; E-Mail: vicente.bernardi@yahoo.com.br; 3 Federal University of Rondônia, Porto Velho, CEP 76801-059, RO, Brazil; E-Mails: katianeguedes@hotmail.com (K.G.B.); luceliabuenoariquemes@hotmail.com (L.B.); 4 Institute of Biophysics Carlos Chagas Filho, Federal University of Rio de Janeiro, CEP 21941-902, RJ, Brazil; E-Mails: rspolti@hotmail.com (R.S.L.); olaf@biof.ufrj.br (O.M.)

**Keywords:** methyl-mercury, fish, pregnancy, fetal growth, gestational age, maternal education

## Abstract

Birth weight can be a predictor of maternal health issues related to nutrition and environmental contaminants. Total hair mercury (HHg) concentration was studied as an indicator of both fish consumption and methylmercury exposure in mothers (and newborns) living in selected low income areas of the Madeira River basin, Amazonia, Brazil. This cohort study (n = 1,433) consisted of traditional riverines (n = 396), riverines who had moved to urban (n = 676) and rural (n = 67) settings, and tin miner settlers (n = 294). Median maternal HHg was significantly different (*p* = 0.00001) between riverine (12.1 µg·g^−1^), rural (7.82 µg·g^−1^), urban (5.4 µg·g^−1^), and tin miner (4.5 µg·g^−1^) groups studied. The same trend (of medians) was observed for newborns’ HHg which also showed significant differences between riverine (3.0 µg·g^−1^), rural (2.0 µg·g^−1^), urban (1.5 µg·g^−1^), and tin miner (0.8 µg·g^−1^) groups. The correlation between maternal and newborn HHg was statistically significant in the riverine (r = 0.8952; *p* = 0.0001), urban (r = 0.6744; *p* = 0.0001), and rural (r = 0.8416; *p* = 0.0001) groups but not in the mother-infant pairs in the tin miner group (r = 0.0638; *p* = 0.2752). Birth weight was significantly different among groups but did not show a pattern consistent with that of fish consumption (and HHg). A multiple regression analysis showed that only family income and gestational age had a significant impact on birth weight. *Conclusions*: Maternal HHg is an important biomarker of maternal fish consumption and of methylmercury exposure during pregnancy. However, in these Amazonian groups, only maternal education and gestational age seemed to affect birth weight positively.

## 1. Introduction

The abundance of fish in the Amazonian ecosystem has been inextricably associated with regional food habits. Amazon fish are the main source of exposure to methylmercury (MeHg)—a neurotoxicant. Environmental mercury occurs naturally in the Amazonian ecosystem [1] and contaminates regional populations that consume the traditionally fish-based diet [2]. This diet exposes vulnerable groups of pregnant mothers and their unborn fetuses to high levels of MeHg. However, lately, the rapid rate of occupation of the Amazon region has brought an increase in immigration and created work opportunities in the newly formed cities and settlements. Slowly but steadily, new demographics and food habits are emerging in the region. 

Total HHg concentration in mothers’ and newborns’ hair was studied in Amazonian urban women with relatively low fish consumption; a significant correlation was found between HHg concentrations in mothers and newborns [3]. Compared to urban mothers, traditional riverine mothers showed significantly higher mercury concentrations in both hair and milk [4]. In these high fish-eating populations, HHg has been shown to be a reliable marker of fish intake and an important measure of MeHg exposure.

Birth weight is central to health issues among them growth and development outcome [5]. Because fish contain essential nutrients, depending on their origin, they can also contain contaminants (which can act as endocrine disruptors). Therefore, both positive and negative associations have been reported for seafood consumption during pregnancy and birth size [6]. Most studies addressing reproductive outcomes have used cord blood as an indicator of Hg exposure; few have used maternal hair-Hg concentrations [7,8,9,10]. Arakawa *et al.* [7] used hair-Hg concentration to study fecundity among Japanese women, while others used it to study birth anthropometry in Poland [8], Austria [9], and France [10]. However, Amazonians habitually consuming freshwater fish have not yet been addressed.

Our recent studies regarding fish consumption (and MeHg exposure) along the Madeira River basin have shown that linear growth of the pre-school children of displaced riverines [11] or of tin mining settlers [12] were not significantly affected. However, birth weight for the city of Porto Velho, in the state of Rondonia, can be affected by smoke from forest fires, frequent in the Amazon [13]. 

The current population living in the Madeira River basin has been impacted by the occupation of the Amazon forest, and food habits are shifting from predominantly subsistence fish-eating to other sources of dietary protein. The objective of this work is to compare current lifestyle choices in the Madeira River basin populations in relation to fish consumption (and MeHg exposure) during pregnancy and evaluate their effect on birth weight.

## 2. Materials and Methods

We have been studying the urban and rural populations of the Madeira River basin for the last 15 years, aiming to assess mercury exposure, health, and development of young children. Lately, our studies have focused on fish-eating habits of current economic and environmental transitioning of the Amazon. The first part of the study was a cross-sectional assessment of health and nutrition status in preschool children from communities of tin miners and former riverines displaced by a hydroelectric dam [11,12]. We extended this work by forming a cohort for a follow-up to monitor growth and neurodevelopment; this also extended to former riverines who had moved to urban areas and non-urban families. The study protocol was approved by the Ethics Committee for Studies in Humans of the Federal University of Rondonia (Of. 001-07/CEP/NUSAU).

The cohort started in January 2006; after contacting pregnant women we obtained their written consent. Participation was voluntary and confidentiality was assured. We provided information guaranteeing that the participant mother could withdraw from the study at any time. The main inclusion and exclusion criteria were (a) residence in the study area for at least five years, (b) being healthy at time of pregnancy (c) and absence of congenital malformations. Information on the delivery condition (home or hospital), gestational age, and birth weight was obtained from hospital records or from midwives. Upcoming publications will address issues related to risk assessment, predictive fetal MeHg exposure (from maternal HHg), neurodevelopment and other health issues.

**Figure 1:**
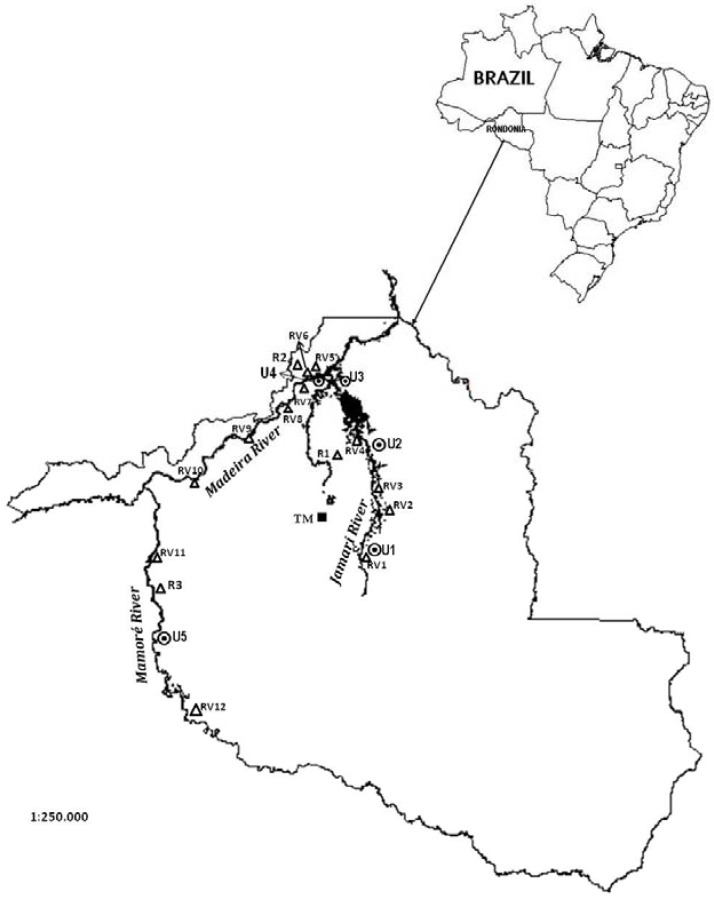
Map of the Madeira River basin illustrating the locations of the studied populations. TM, tin miners. **Δ Rural and riverine communities:** RV1, Marechal Rondon; RV2, Comunidade do Rio Preto do Crespo; RV3, Rei do Peixe; R1, Triunfo; RV4, Vila dos Pescadores; RV5, São Sebastião; R2, Vila Jatuarana; RV6, Engenho Velho; RV7, Santo Antonio; RV8, Teotônio; RV9, Jaci-Paraná; RV10, Mutum-Paraná; RV11, Vila Murtinho; R3, Vila Iata; RV12, Vila Surpresa. ◉ **Urban sampling sites.** U1, Ariquemes; U2, Itapuã; U3, Candeias; U4, Porto Velho; U5, Guajará-Mirim.

The sampling sites are marked on the map in Figure 1, which shows an area running more than 733 km along the Madeira River basin. We invited 1,668 pregnant mothers; 215 declined to participate in the study, 11 miscarried, and nine were excluded because their children were born with congenital anomalies (neural tube defects and gastroschisis). Altogether, we surveyed 1,433 mother-newborn pairs from nine communities encompassing different economic activities: (a) riverines (n = 396), formed by families still depending heavily on the river for their sustenance (communities along the banks of the Jamari, Madeira and Mamoré Rivers, areas—*RV1 to RV12*); (b) urban (n = 676), characterized mostly by former riverines now living on the outskirts of Ariquemes (*U1*), Itapuã (*U2*), Candeias (U3), Porto Velho (*U4*), and Guajará-Mirim (*U5*); (c) rural (n = 67)—*R1 to R3*; (d) tin miners (n = 294) (*TM*), former itinerants (a mobile population that comes and goes as a result of the price of cassiterite—tin ore). The tin-mining families are considered a contrasting group regarding low fish consumption and exposure to a mining environment. These families are mostly from out of state, with different food preferences (which do not include fish as the main dietary source of protein) and source of income. The groups of riverines, rural, and urban dwellers share a traditional habit of eating fish and regional foods (mostly provided by the forest). Besides that, tin miners are also exposed to hazardous contaminants resulting from metal extraction and processing. Although we did not measure background concentrations of toxic metals, it is expected that these mothers are also exposed to some MeHg resulting from fish consumption but less than the other groups [12].

### 2.1. Data Collection

The protocol for data collection and questionnaires was detailed in preceding publications [11,12]. Trained interviewers administered the questionnaire to the mothers, addressing family characteristics (number of children), income and resources, housing facilities, hygiene, and fish-eating habits. Fish consumption questionnaires were the same as those used in previous studies and were all properly validated. Each family was visited and interviewed on a single occasion within the first week of puerperium. Birth size (weight, length, and head circumference) was obtained from hospital records. For mothers that chose to deliver at home, we provided midwives with a scale and measuring tape to record the anthropometric data.

Mothers were asked how many days fish was served during the week, and a hair sample was cut from both mother and newborn using stainless steel scissors. The hair sample was cut from the back of the head (always in the same occipital area) close to the scalp, bundled together, and placed in a labeled envelope. Total Hg determination was done at the Radioisotopes Laboratory of the Federal University of Rio de Janeiro, according to routine procedures described previously [3]. The method involves sample digestion followed by reduction to elemental Hg vapor. Briefly, hair samples were washed with EDTA 0.01%, dried in an oven at 50 °C, weighed, and digested with 5 mL of HNO_3_:H_2_SO_4_ (1:1) and 4 mL of 5% KMnO_4_ using a digestion block at 80 °C for 40 min. Total Hg in the digested samples was done by cold vapor atomic absorption spectrometry with a flow injection system-FIMS (CV-AAS; Perkin-Elmer-FIMS 400, Ueberlingen, Germany). 

### 2.2. Statistical Analysis

We tested for the data normality and non-normal distribution using the Kolmogorov-Smirnov one-sample test in order to apply appropriate correlation and hypothesis tests. The relationships between the number of fish servings consumed by mothers and the HHg concentration in maternal hair and between maternal and newborn HHg concentrations were studied using Spearman correlation analysis with all samples; in this analysis we considered meals as a categorical variable. In the null-hypotheis test and linear regression models we used only the variables that had a complete set of data, *i.e.*, there were 133 cases of missing data in family income. The variables (maternal education, gestational age, maternal age, number of pregnancies, family income, infant and maternal hair-Hg) included in the regression model were based on known effects on health and birth outcome. Maternal education entered as a discrete variable, number of years spent at school.

Group medians were compared for maternal (HHg concentrations, days of fish consumption) and newborn variables using a Chi-square test and *post-hoc* multiple comparisons of mean ranks for all groups after Bonferroni test adjustment [14]; for the sake of representation we used the least acceptable level of significance (*p* < 0.05). Statistical analyses were done with XLSTAT (Adinsoft, version 1.01, 2013, Paris, France). Multiple linear regression analysis (casewise) was used to assess the relationship between birth weight and fish consumption (as maternal and newborn hair-Hg) as well as maternal age, number of siblings, family income, maternal schooling. We ran the regression model with cohorts (groups) as random factor to assess potential interactions of predicting variables of birth weight. 

## 3. Results

The map in Figure 1 illustrates the study areas that formed the riverine (RV1 to RV12), urban (U1 to U5), rural (R1 to R3), and tin miner (TM) groups. In Table 1 the summary of the maternal and newborn studied variables is shown. Most maternal variables showed statistically significant differences between group medians. Birth weight was statistically different between groups; however, the Bonferroni test adjustment showed statistical significance only between rural and tin miner. riverine and tin miner groups showed no statistically significant differences in birth weight but showed striking differences in fish consumption (Table 1).

**Table 1:** Summary of maternal constitutional (age, gestational age, and number of pregnancies) and environmental (family income, number of school years, hair-Hg concentrations) mother-newborn (anthropometry, hair-Hg concentrations) pairs of low-socioeconomic families inhabiting the Madeira River basin.

Characteristics	Riverine (n = 396)	Urban (n = 676)	Rural (n = 67)	Tin mining (n = 294)	*p* (Median Test)
	*Median (min–max)*	*Median (min–max)*	*Median (min–max)*	*Median (min–max)*	
*Mothers*					
Hair-Hg (µg·g^−1^)	12.12 (1.02–130.72) ^a^	5.36 (0.73–24.14) ^b^	7.82 (2.56–41.1) ^ac^	4.45 (1.53–11.94) ^d^	0.00001
Fish serving, w	5 (0–7) ^a^	2 (0–7) ^b^	3 (2–7) ^c^	1 (0–2) ^d^	0.00001
Income *	450 (100–2,000) ^a^	600 (100–4,500) ^b^	450 (50–1,500) ^ac^	600 (70–2,500) ^d^	0.00001
Mother education, year	4 (0–15) ^a^	7 (0–16) ^b^	5 (0–15) ^ac^	6 (0–16) ^cd^	0.00001
Age, year	20 (13–41) ^a^	22 (13–42) ^b^	21 (16–42) ^abc^	22,5 (13–43) ^cd^	0.0116
Pregnancy, n	2 (0–12) ^a^	2 (0–8) ^ab^	2 (0–10) ^abc^	2 (0–5) ^abcd^	0.0101
*Newborns*					
Weight, g	3,150 (2,010–5,250) ^a^	3,215 (2,200–5,950) ^ab^	3,010 (2,040–4,350) ^ac^	3,200 (2,200–4,500) ^abd^	0.0482
Length, cm	50 (44.5–59) ^a^	51 (43–59.5) ^ab^	50 (43–56.5) ^abc^	51 (44.5–57.5) ^abcd^	0.0218
Gestation age, week	39 (35–43) ^a^	39.5 (35–43) ^ab^	39 (35–42) ^abc^	39 (32–43) ^cd^	0.00001
Hg (µg·g^−1^)	3.01 (0.09–18.53) ^a^	1.5 (0.11–4.81) ^b^	1.98 (0.29–8.77) ^c^	0.80 (0.12–1.99) ^d^	0.00001

* Per month in Real (national currency). Medians with different superscript letters are statistically significant after Bonferroni test adjustment (*p* < 0.05).

**Figure 2:**
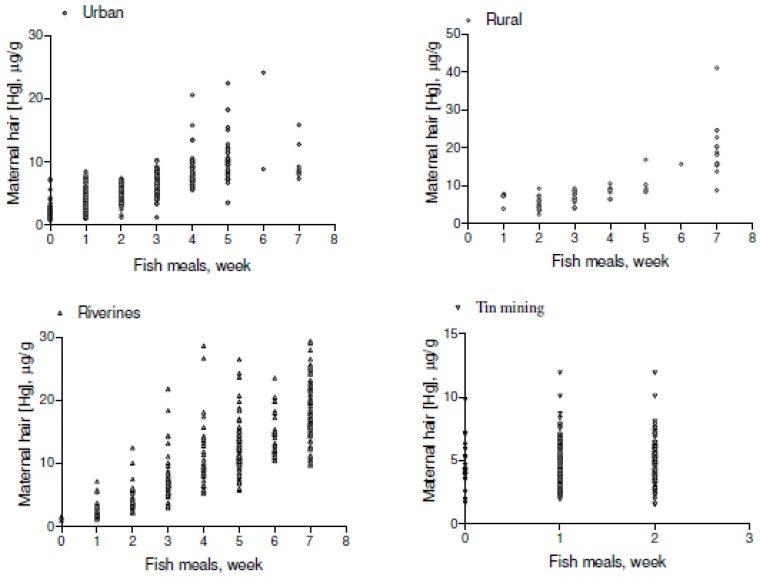
Scatter plot of total hair-Hg concentrations as a function of maternal fish consumption.

Most of the biological variables for newborns showed a statistically significant difference between groups. The median variation in newborns’ HHg showed a pattern similar to that seen in mothers’ HHg. Newborns of riverine mothers also showed the highest HHg concentrations. When comparing fish consumption among the 4 groups, the median number of servings per week differed significantly between each group (*p* = 0.00001). For these mothers there was a significant correlation between fish consumption and total HHg concentrations in riverine (Spearman r = 0.8089, *p* = 0.001), urban (Spearman r = 0.7681, *p* = 0.001), rural (Spearman r = 0.7644, *p* = 0.001), and tin miner (Spearman r = 0.1211, *p* = 0.001) groups (Figure 2). The relationship between fish consumption and maternal HHg is shown in the scatter-plot in Figure 2.

**Figure 3:**
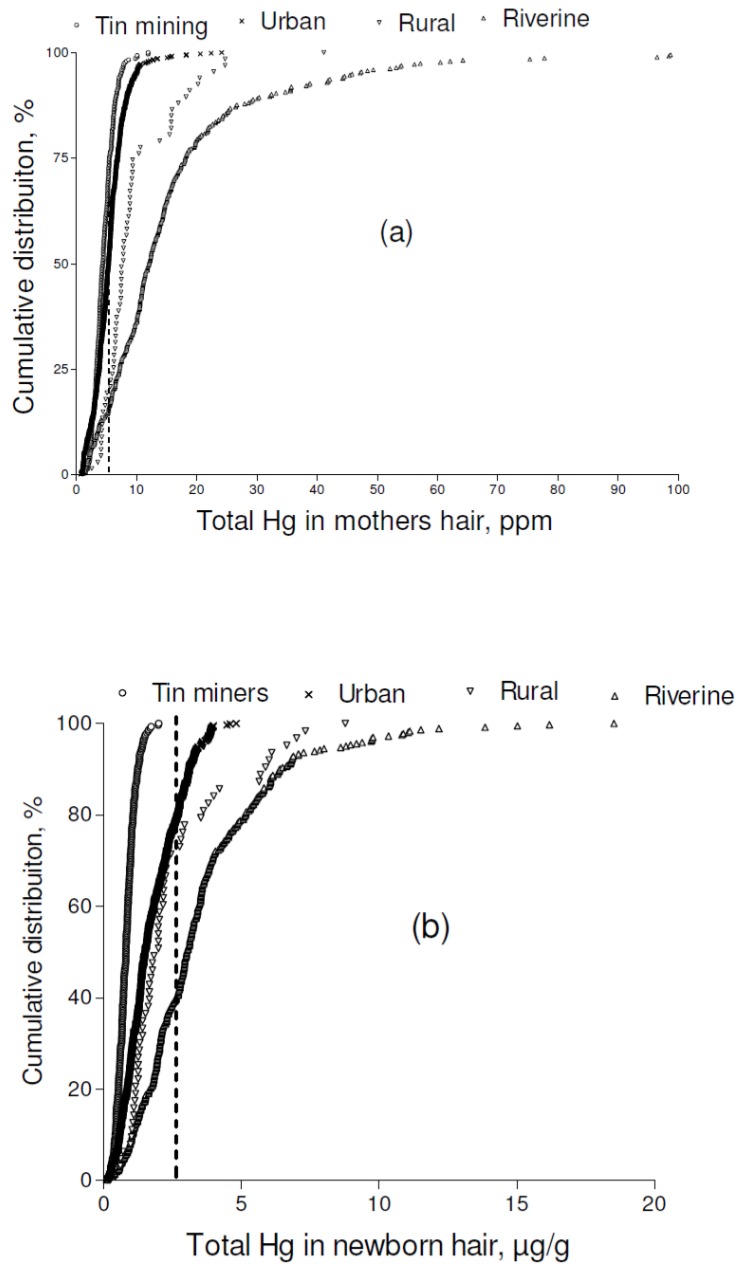
Cumulative frequency distribution of total hair-Hg concentrations in studied communities of the Madeira River Basin representing mothers (**a**) and infants (**b**).

The cumulative percent distribution of HHg in mothers and newborns is shown in Figure 3. The proportion of HHg below 2.5 µg·g^−1^ was clearly small for all groups (urban 12%; rural = 0%; riverine = 6%; tin miner = 8%); except for Tin miner, overall, the majority (more than 50%) of the HHg concentrations were above the recommended level of 5.0 µg·g^−1^ (urban 56%; rural = 81%; riverine = 86%; tin miner = 38%). The HHg distribution in newborns was similar to that seen in their mothers, who had circa fourfold higher HHg concentrations. 

**Figure 4:**
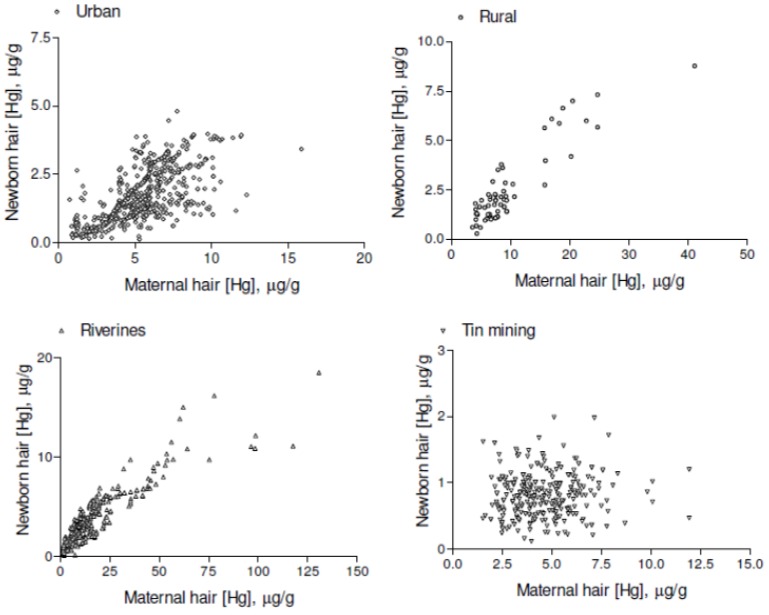
Scatter plots of total hair-Hg concentrations of mothers and respective newborns.

Correlations between maternal and newborn HHg are illustrated by the scatter-plot in Figure 4. Maternal and newborn HHg correlations were statistically significant in riverine (r = 0.8952; *p* = 0.0001), urban (r = 0.6744; *p* = 0.0001), and rural (r = 0.8416; *p* = 0.0001) groups but not in the mother-infant pairs in the tin miner (r = 0.0638; *p* = 0.2752) group.

A summary of the multiple regression analysis is shown in Table 2. Gestational age and maternal education were the only variables seen to significantly influence birth weight.

**Table 2:** Summary of multiple regression of chosen variables that might influence birth weight.

	β1 *	SE β1 *	β0	SE β0	t (1,291)	*p*-value
Intercept			131.173	307.720	0.4263	0.700
Gestation age, w	0.274	0.027	79.682	7.795	10.222	0.0001
Age, year	−0.006	0.039	−0.449	3.024	−0.148	0.882
Pregnancy, n	−0.036	0.038	−9.996	10.607	−0.942	0.346
Newborn Hg (µ·g^−1^)	−0.069	0.052	−17.745	13.301	−1.334	0.182
Mother Hg (µ·g^−1^)	0.033	0.052	1.514	2.407	0.629	0.529
Income *	−0.028	0.030	−0.030	0.032	−0.937	0.348
Mother education, year	0.078	0.030	11.740	4.551	2.579	0.010

SE, standard error; * Per month in Real (national currency).

## 4. Discussion

This study is unique in that addresses differences in subsistence freshwater-fish consumption (as HHg exposure) in relation to birth weight. The results clearly show differences in freshwater-fish consumption among groups. In the Madeira River basin, riverines and rural inhabitants still maintain high fish consumption. Due to the remote nature of their location they had the lowest schooling and family income, but the highest fish consumption and HHg concentrations. However, significant differences in birth weight were not influenced by a proxy of fish consumption (hair-Hg). Overall, the majority of mothers in the riverine and rural settings consumed fish caught as part of their livelihood, while for the urban and tin miner mothers, fish were mostly acquired from local markets according to affordability. 

Birth weight was nevertheless influenced by gestational age and maternal education. Birth weight can be influenced by a variety of constitutional and environmental factors, and maternal seafood intake has been reported to have positive, negative, or no effect (see references in Dórea [15]). In respect to studies of maternal seafood-consumption, non-concurrent results of birth weight outcomes were attributed to types of seafood (fish and shellfish) or contaminants [6]. Although Amazon basin freshwater-fish can contain environmental contaminants [16], results of our study indicate that modifying factors other than fish consumption (and Hg exposure) may play a more influential role in birth weight.

Studies relating maternal HHg and birth weight have been conducted in European countries where a variety of seafood is consumed. Sikorski *et al.* [8] reported an inverse relationship between birth weight and maternal HHg concentration in Poland. However, no significant association between birth weight and maternal HHg levels has been reported in Austria [9], or in France [10]. In these studies the levels of HHg were far below those of the women from the Madeira River basin. Indeed, HHg concentrations in women in reproductive age groups in 17 European nations showed that for most countries the proportion of women attaining levels higher than 2.5 µg/g [17] was much lower than among mothers in our study.

Birth weight is a direct function of fetal growth which, among other things, depends on gestational age [18]. As expected, our results are concurrent with the literature, and they also agree with the observed trend that in a low socioeconomic environment maternal education has a positive effect on fetal growth [19,20,21,22]. 

It is clearly shown that maternal HHg concentration is related to dependence on fish consumption. Indeed, direct fish consumption can be assessed by HHg (Figure 2, Figure 3). Correlation analysis confirms that HHg, in the Amazon, is a biomarker of fish intake and the reported strong coefficients support the rationale of a direct relationship between HHg and fish intake. For the high fish-consuming groups (riverine, urban, and rural communities) the correlations of maternal HHg and newborn HHg were all highly significant. These results concur with studies relating to HHg seafood consumption in European countries [8,9,10] and Japan [23].

Mean fetal Hg accumulation (represented by newborn HHg) in these Amazonian populations is higher than that reported for babies of European mothers exposed to dental amalgam fillings [24,25], and seafood [8,26], as well as being higher than in urban Amazonians consuming freshwater fish [3,27], or urban Japanese mothers [28]. It is worth mentioning that the ratios of maternal to fetal HHg in all these studies were quite distinct in magnitude. Except for two studies [27,28], in most of them, including ours, maternal HHg was always higher than newborn HHg. 

Fish is the best single-food source of essential nutrients habitually consumed by Amazonians. High fish consumption has placed these populations in the category with the highest levels of HHg concentrations [2]. However, current dietary habit in the present socioeconomic and demographic situation has shifted fish dependence to other nutritional sources. Although Amazonian fish can contain omega-3 polyunsaturated fatty acids (PUFA; decosahexanoic [22:6] acid (DHA) and eicosapentaenoic [20:5] acid) in much lower concentrations [29] than marine fish, we could not see birth weight associated with differences in freshwater fish consumption. 

Fresh-water fish is also the most important source of MeHg for humans in the Brazilian Amazon. Taking into account compensating components (against MeHg toxicity) in freshwater-fish and sea-food, the World Health Organization (WHO) proposes a tolerable daily limit of mercury exposure corresponding to a hair-Hg concentration of 2.5 μg/g [30]. Despite declining fish-eating habits in the Western Amazon, the studied groups still showed relatively high levels of HHg. Compared to previous studies on riverine mothers from the Madeira River basin our results are slightly lower [31,32]; however, HHg concentrations remain remarkably close to those of urban mothers [3].

This study is the largest one that has assessed mother-newborn pairs in the Brazilian Amazon, which constitutes its main strength. Additionally, maternal HHg, a proxy of fish consumption, was reliably measured, and fetal mercury exposure (a difficult parameter to measure) could be assessed by newborn HHg. Although we captured current fish consumption through HHg (Figure 2, Figure 3), one of the limitations of this paper is that we could not compare fish-replacement foods among groups. In addition, we could not account for circumstances where a higher MeHg exposure (HHg) might contribute to differences in birth weight. 

## 5. Conclusions

Maternal HHg is an important biomarker of maternal fish consumption and of methylmercury exposure during pregnancy. However, in these Amazonian groups, only maternal education and gestational age seemed to positively affect birth weight.
